# Development of a core outcome set for the evaluation of shared decision-making interventions in healthcare: a study protocol

**DOI:** 10.1136/bmjopen-2026-116623

**Published:** 2026-09-15

**Authors:** Christin Hoffmann, Joanne E Butterworth, Florian Naye, Maureen Smith, Taona Nyamapfene, Samuel Lawday, Kerry Avery, Hilary Bekker, Paulina Bravo, Simon Décary, Adrian Edwards, Glyn Elwyn, Ellen G Engelhardt, Juan Victor Ariel Franco, Mirjam M Garvelink, Anik MC Giguere, Martin Härter, Tammy Hoffmann, Simone Kienlin, Kirsten McCaffery, Janneke Noordman, Karina Olling, Lilisbeth Perestelo-Pérez, Arwen H Pieterse, Fülöp Scheibler, Karen Sepucha, Dirk Ubbink, KD Valentine, Robert J Volk, Felix Wehking, Sang Ho Yoo, Helen Bulbeck, Amy C Cole, Maarten de Wit, Jeanette Finderup, Katie Geary, Christine M. Gunn, Wen-Hsuan Hou, Ashley J Housten, Min Ji Kim, Marti Norma, Lissa Pacheco-Brousseau, Lisbeth Snede, Cindy Tian, Karine Toupin-April, Angus GK McNair

**Affiliations:** 1 NIHR Bristol Biomedical Research Centre, Bristol, UK; 2 University of Exeter, Exeter, UK; 3 Université Laval, Québec City, Quebec, Canada; 4 Patient Partner, Ottawa, Ontario, Canada; 5 University Hospitals Plymouth NHS Trust, Plymouth, UK; 6 Leeds Institute of Health Sciences, University of Leeds, Leeds, UK; 7 Fundación Arturo López Pérez and Universidad Finis Terrae, Santiago, Chile; 8 University of Sherbrooke, Sherbrooke, Quebec, Canada; 9 Cardiff University, Cardiff, UK; 10 Dartmouth College, Hanover, New Hampshire, USA; 11 Epidemiology, The Netherlands Cancer Institute, Amsterdam, Netherlands; 12 Heinrich Heine University Düsseldorf, Düsseldorf, Germany; 13 Value-Based Healthcare, Sint Antonius Hospital, Nieuwegein, Netherlands; 14 University Medical Center Hamburg-Eppendorf, Hamburg, Germany; 15 Bond University, Gold Coast, Queensland, Australia; 16 South-Eastern Norway Regional Health Authority, Hamar, Hedmark, Norway; 17 The University of Sydney, Sydney, New South Wales, Australia; 18 Netherlands Institute for Health Services Research, Utrecht, Netherlands; 19 Center for Shared Decision Making, Vejle-Lillebaelt University Hospital of Southern Denmark, Veijle, Denmark; 20 Canary Islands Health Service: Servicio Canario de Salud, Tenerife, Spain; 21 Biomedical Data Sciences, Leiden University Medical Center, Leiden, Netherlands; 22 National Competency Center for Shared Decision Making, University Hospital Schleswig-Holstein - Campus Kiel, Köln, Germany; 23 Harvard Medical School, Boston, Massachusetts, USA; 24 Surgery, Amsterdam University Medical Centres, Amsterdam, Netherlands; 25 MD Anderson Cancer Center, The University of Texas, Houston, Texas, USA; 26 Department of Emergency Medicine, Jena University Hospital, Jena, Thuringia, Germany; 27 Hanyang University, Seongdong-gu, Korea (the Republic of); 28 University of Exeter, Patient and public representative, Exeter, UK; 29 University of North Carolina at Chapel Hill School of Medicine, Chapel Hill, North Carolina, USA; 30 Patient and public representative, Zaltbommel, Netherlands; 31 Department of Clinical Medicine, Aarhus Universitet, Aarhus, Central Denmark Region, Denmark; 32 University of Warwick Medical School, Coventry, UK; 33 Taipei Medical University Hospital, Taipei City, UK; 34 Washington University School of Medicine, Washington, District of Columbia, USA; 35 Patient and public representative, North Carolina, North Carolina, USA; 36 University of Ottawa, Ottawa, Ontario, Canada; 37 PiCC United (Patient Involvement & Collaboration Community United), Denmark, Denmark; 38 The Chinese University of Hong Kong, Hong Kong, Hong Kong

**Keywords:** Patient Participation, Patient Reported Outcome Measures, Community Participation, Health Services

## Abstract

**Abstract:**

**Introduction:**

Shared decision-making (SDM) is a process whereby patients are supported to reach decisions about their healthcare in collaboration with healthcare professionals. Despite decades of SDM research, the impact of using SDM interventions (eg, patient decision aids, decision coaching, question prompt lists, training and feedback or service changes) on clinical and health service outcomes remains uncertain. This is partly because synthesis of the existing literature is hindered by substantial heterogeneity in the evaluation of interventions to facilitate SDM. A core outcome set (COS) is an agreed standardised set of outcomes that should be measured and reported in all effectiveness studies. The aim of this study is to develop a generic COS for evaluating the impact of SDM interventions (COS-SDMi).

**Methods and analysis:**

This study will follow Core Outcome Measures in Effectiveness Trials (COMET) handbook and Core Outcome Set-STAndards for Development (COS-STAD) and reporting (COS-STAR). Relevant key interest holder groups include health professionals, patients, academic experts in SDM, representatives from policy/guideline development, journal editors and research funders. The scope of the COS was agreed through consultation with experts from key interest holder groups. The study will consist of three steps. In Step 1, we will produce a ‘long’ (comprehensive) list of candidate outcome domains using multiple data sources. Relevant outcomes will be extracted from a COS developed in the context of rheumatology, a synthesis of existing reviews of SDM interventions and qualitative interviews with a minimum of 20 representatives of key interest holder groups internationally. In Step 2, we will agree on a shorter list of outcome domains using a Delphi survey. A sequential two-round international online Delphi survey with two panels (professionals, public/patients) will be used to agree on the outcomes, seeking responses from a minimum of 150 international participants representing key interest groups. In Step 3, we will reach consensus on the final COS using consensus meetings. Consensus meetings with key interest holder group participants internationally will be held, applying modified nominal group techniques. The total project period will be from June 2025 until September 2026.

**Ethics and dissemination:**

Research ethics approval has been granted in the UK for undertaking qualitative interviews (University of Exeter Faculty Ethics Committee, ref: 8207624) and the Delphi survey and consensus meeting activities (University of Bristol Faculty Ethics Committee, ref: 7741). Written informed consent will be obtained digitally from all participants in the qualitative interviews, the Delphi surveys and the consensus meetings. The final COS will be disseminated through presentations at international conferences, briefing notes to key organisations and publication in a peer-reviewed journal. Further dissemination is planned through our patient/public advisory group, professional networks and executive group channels, as well as publications to patient groups, funders, journal editors, international regulatory bodies and health technology assessment boards.

**Study registration:**

COMET Database (www.comet-initiative.org/Studies/Details/3586).

**STRENGTHS AND LIMITATIONS OF THIS STUDY**

- The core outcome set development follows established guidelines and best-practice recommendations (Core Outcome Measures in Effectiveness Trials (COMET), Core Outcome Set-STAndards for Development (COS-STAD) and Core Outcome Set-STAndards for Development and reporting (COS-STAR).
- The study draws on international and multi-interest holder participants and includes clinical and non-clinical disciplines and patient partner organisations.
- The broad scope of the core outcome set and inclusion of a range of interest holder participants may introduce heterogeneity in perspectives and priorities that require careful navigation and transparent reporting.
- Delphi survey and consensus meetings are conducted in English, which limits the relevance to non-English speaking contexts.

## Introduction

### Background and context

 Shared decision-making (SDM) is a process where patients are supported to reach decisions about their healthcare in collaboration with healthcare professionals.^1^ SDM involves patients, professionals and others working together to choose care based on scientific evidence, clinical expertise and personal preferences and values. International policy,^2 3^ professional and regulatory guidelines^4 5^ recommend SDM for a wide range of healthcare decisions in a variety of clinical contexts. SDM is recognised as a patient’s right, has an ethical imperative and inherent social value.^3 6^ Individuals often report that the process of SDM is a meaningful experience.^7^

Several types of interventions are designed to help facilitate the SDM process. These SDM interventions take a variety of practical applications, including, but not limited to, patient decision aids, decision coaching, question prompt lists, SDM training and feedback and healthcare service changes.^8^ They may target one or more individuals who can be viewed as acting at various levels of a patient-centred healthcare system (ie, interventions may work by changing the behaviours of patients themselves or those of healthcare professionals, service managers, commissioners or policy makers). Some SDM interventions are complex and may have multiple interacting components, targeting more than one individual acting at multiple levels of the healthcare system and impact several outcomes.^9^

Health technology assessment (HTA) is the process to evaluate the impact of health interventions and inform evidence-based decision-making in healthcare systems by assessing value beyond efficacy alone.^10 11^ High-quality HTA therefore requires an understanding of how interventions designed to facilitate the adoption of SDM influence clinical and health service outcomes, extending beyond process and patient experience.

Potential outcomes of these different SDM interventions range from those that assess the SDM process (including patient and professional experiences) through those that assess the effect of an improved SDM process on patient, healthcare professional and system, including short and longer-term outcomes. Outcomes reported in the existing literature to evaluate the effectiveness of SDM interventions include patient knowledge of available options and informed preferences, realistic patient expectations about their care, decision self-efficacy, decisional conflict, collaborative deliberation between patient and professional, alignment between a chosen management option and a patient’s values in the context of their daily lives, impact of chosen option on patients, improved patient self-management, use of treatments with greater benefit-to-harm ratios, preference for the most intensive management plans and healthcare utilisation.^12–25^

Some of these outcomes may influence another. Evidence is emerging to enable the evaluation of mechanisms underlying the components of SDM interventions, to enable the mapping of non-linear, interacting pathways from process through to outcomes.^26^ Some evidence suggests that there might be longer-term impacts on health services. SDM interventions may reduce unwarranted practice variation and costs by reducing the rate that patients opt for unnecessary, expensive treatments.^27^ A systematic review identified 15 studies (11 randomised controlled trials; 10 low risk of bias) that evaluated the impact of decision aids on these outcomes.^28^ Three demonstrated statistically significant reductions in rates of prostatectomy, hip/knee replacement and hysterectomy. This suggests that decision aids can support patients in making informed, values-based treatment decisions, with the observed reduction in surgery rates likely reflecting that some patients, when better informed about their options, choose more conservative treatments that align with their preferences.^29^ This may have implications for healthcare resource use and costs through a lower number of surgical procedures performed. There may also be downstream implications for procedure-related risks and consequences through avoiding complications and healthcare burden and costs for patients.

### Rationale

Despite decades of research on SDM, the effect of SDM interventions on outcomes relating to patients, professionals, organisations, health systems and populations remains largely uncertain. The conduct of evidence syntheses/meta-analyses assessing the impact of SDM interventions is hindered by (i) lack of differentiation between SDM process measures and outcome measures when evaluating SDM interventions, (ii) lack of consensus on the core outcomes of SDM interventions^30^ and (iii) lack of transparency when selecting these outcomes. These limitations result in heterogeneity of outcomes and research waste. There is a need to develop a core outcome set (COS) to evaluate SDM interventions in the most optimal manner in primary studies, thus reducing the heterogeneity of outcomes and research waste and facilitating the conduct of evidence syntheses/meta-analyses.

Efforts have been made to address these problems through the development of a COS for use when evaluating SDM interventions in rheumatology (hereafter defined as the rheuCOS-SDMi).^30^ A COS is an agreed standardised set of outcomes that should be measured and reported, as a minimum, in all comparative effectiveness studies in specific areas of health or healthcare.^31^ The rheuCOS-SDMi was co-developed with significant input from patients, clinicians and SDM experts in the field of rheumatology, using accepted methods to gain consensus on the COS within a rheumatology setting. It does, however, have some limitations. Initial development lacked clear conceptualisation to differentiate between SDM process and outcome assessment, although this was later clarified. The generation of a ‘long’ (comprehensive) list of outcome domains predated relevant, comprehensive systematic reviews^32–34^ that may have subsequently identified outcome domains to be shortlisted for inclusion in the consensus process. It is unclear whether the domains identified within a rheumatology context are relevant, comprehensive, or comprehensible when applied in other healthcare contexts, and therefore whether the rheuCOS-SDMi is transferable to the assessment of a variety of SDM interventions tailored to and interacting with a range of patient populations, healthcare settings and contexts.

Use of SDM interventions across diverse contexts is likely to require tailoring and adaptation of SDM processes through the utilisation of intervention components that interact with the populations and settings in which they are delivered.^8 35^ For example, different clinical contexts may require different types of healthcare decisions to be made or bring additional complexity into the patient-practitioner interaction (eg, for patients who live with multiple long-term conditions). There may be differences in pathways of care and different timeframes for decision-making because of variation in healthcare organisation. There may be differences in societal or cultural expectations and differences in resource provision because of variation in healthcare policy. All of these contextual factors influence the use of the components of SDM interventions, as well as any assessment of their effectiveness.

### Aim

The aim of this study is to develop a COS to evaluate SDM interventions. The COS will be applicable across diverse populations and healthcare contexts, to all types of SDM interventions targeting individuals acting at various levels of a patient-centred healthcare system. The COS will be relevant to patients and caregivers, healthcare professionals, health services and systems and wider society. The COS will support consistent outcome selection, reduce reporting heterogeneity and enable more robust syntheses and economic analyses of SDM interventions. This will enable meaningful health technology appraisal and impact on healthcare policy. We define this new COS as the COS-SDMi.

## Methods and analysis

### Study design

**Figure 1:**
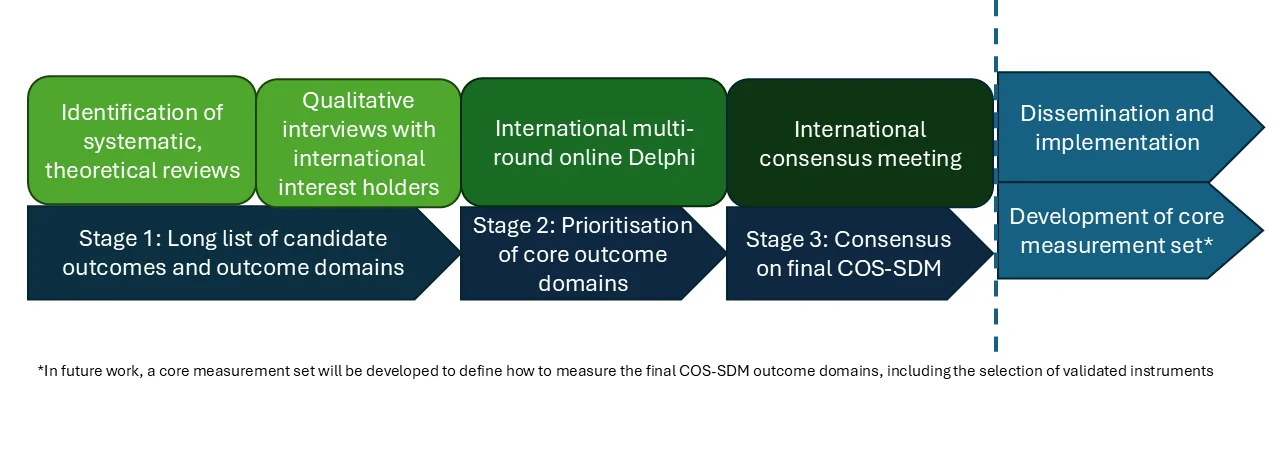
COS for use when evaluating SDM interventions (COS-SDMi) study flowchart.

This study will adhere to methods for COS development outlined in the Core Outcome Measures in Effectiveness Trials (COMET) handbook and Core Outcome Set-STAndards for Development (COS-STAD) guidelines^32 36^ and follow guidelines for the reporting of COS development studies (COS-STAR).^37^ See figure 1 for the study flowchart. Key steps will be as follows:

- Step 1: Production of a long list of candidate outcome domains using multiple data sources, including evidence syntheses and qualitative methods.
- Step 2: Rating of the essentiality of outcome domains using a sequential two-round online Delphi.
- Step 3: Consensus on the final COS through consensus meetings with modified nominal group technique.

This project has been registered in the COMET Database (www.comet-initiative.org/Studies/Details/3586). The total project period will be from June 2025 until September 2026.

#### COS scope

The scope of the COS-SDMi (see box 1) was defined through consultation with a panel of international, multidisciplinary interest holders relevant to a broad range of healthcare contexts (including experts in SDM, healthcare professionals, public/patient partners). Consultations included two online meetings with structured discussions.

Box 1 **The scope of the COS-SDMi**

**Health conditions**—All health conditions and adult populations who are involved in healthcare decision-making. This can therefore include decisions related to undertaking tests and procedures (eg, used in screening and diagnosis), making lifestyle choices (eg, stopping smoking, using physical activity programmes), considering treatment (eg, medications, surgery, physiotherapy, counselling) or for advanced care planning. The COS will be inclusive of decision-making in maternity care, while recognising that pregnancy may not be considered a health condition, and in situations where there is the need for important others (eg, carers or relatives) as proxy decision-makers (eg, where another person participates in SDM on behalf of a patient who is not cognitively able to do so).

**Interventions**—All types of interventions aimed at facilitating SDM in clinical and non-clinical contexts, with components that may target patients/public, health and allied health professionals, patient pathways within health systems or have a combination of interacting components. Examples of interventions include patient decision aids, decision coaching, question prompt lists, training and feedback programmes for healthcare providers, educational programmes for patients, service changes designed to support patients and/or clinicians during the decision-making process or any combination of these. (This list is not exhaustive.)

**Context of use**—The COS will be recommended for use in clinical and non-clinical comparative effectiveness research (eg, randomised controlled trials) and observational, ie, non-interventional, studies.

COS, core outcome set; SDM, shared decision-making.

##### Key interest holder groups

The following interest holder groups will be involved in the COS development process:

- Patients, carers, public members, patient organisations and patient advocacy groups representing lived experiences of a wide range of healthcare decision-making.
- Healthcare and allied health professionals (eg, doctors, nurses, dentists, dieticians, occupational therapists, paramedics, physiotherapists, psychologists, healthcare educators) involved in the care or decision-making process that includes patients with a wide range of healthcare conditions.
- Academic experts in SDM (eg, trialists, educators, decision scientists, developers of SDM interventions and/or measurement instruments), ethics, law, outcomes methodology, implementation science and health economics.
- Representatives and decision-makers from policy, guideline development, HTA, commissioning bodies or global non-governmental organisations.
- Journal editors.
- Research funders.

### Patient and public involvement

Patient and public involvement is central to the development of the COS-SDMi. A patient partner (MS) serves as a co-investigator and member of the study delivery team to ensure that patient perspectives remain integral throughout. An international patient/public advisory group (PPAG), comprising patient/public partners from their respective countries, has been convened to provide input at various key stages of the project. MS (with support from the study management team) will lead the PPAG activities, monitor progress against milestones, help shape the conduct of consensus activities, and serve as a key link to the executive group by taking part in executive group meetings. The PPAG have already shaped the design of this project. Individual PPAG members have reviewed and co-authored this protocol. The PPAG will continue to be involved throughout the planning, design, conduct, and dissemination of this study. The PPAG will have significant input and oversight during the development of and recruitment for consensus activities. For example, the group will review outcome domains for relevance and comprehensiveness and assess the Delphi survey for clarity and comprehensibility. Their expertise will be sought when disseminating the questionnaires, supporting recruitment through their international networks and organisations. All patient and public-facing materials will be co-developed with PPAG input. The perspectives of the PPAG will be given equal weighting to the perspectives of the executive group during interpretation of data, to inform decision-making by the project co-leads and the study delivery group. All PPAG activities and the impact that they have on the study will be captured in an Impact Log and reported.

### Step 1: Production of a ‘long list’

A ‘long’ (comprehensive) list of candidate outcomes and outcome domains will be produced from multiple data sources, including evidence syntheses and qualitative methods.

A total of three data sources will be used: (i) an existing COS developed in the context of rheumatology, (ii) existing reviews of SDM interventions and (iii) qualitative interviews.

#### Existing COS developed in the context of rheumatology

An initial ‘long list’ of candidate outcomes and outcome domains will be developed by extracting the outcome domains included in the rheuCOS-SDMi that was developed using four different data sources (box 2), including a robust Cochrane systematic review.

Box 2 **Data sources used to develop rheuCOS-SDMi**

- Systematic review of SDM domains.
- Cochrane systematic review of outcomes of studies evaluating patient decision aids.
- Interviews with patients/professionals in a rheumatology context.
- Survey of patients/professionals in a rheumatology context.
- SDM, shared decision-making.

#### Existing reviews of SDM interventions

Targeted searches will be conducted to supplement the initial long list with additional relevant outcomes and/or outcome domains. There already are a high number of contemporaneous, high-quality evidence syntheses of SDM studies which are known to the authors a priori (box 3), including two further Cochrane reviews. These will be reviewed to extract further unique candidate outcomes.

Box 3 **Existing reviews of SDM interventions and their outcomes identified a priori**

- Cochrane review of interventions to increase the use of SDM (Legare *et al*^8^).
- Cochrane systematic review of decision coaching for people making healthcare decisions (Jull *et al*^48^).
- Scoping review of long-term outcome of SDM on patients, professionals and health services (Wehking *et al*^49^).
- Scoping review of decision aids for health promotion (Gultzrow *et al*^50^).
- A framework for conducting economic evaluations when using patient decision aids in healthcare decision making (Ara *et al*^51^).
- SDM, shared decision-making.

Additional existing reviews of SDM interventions will be identified through applying the keywords ‘shared decision making’, ‘outcomes’, ‘review’ and ‘impact’ to the Cochrane Library database of systematic reviews and Google Scholar. Records will be screened and included if they: (i) report a systematic, scoping or narrative review, (ii) describe the effects of SDM interventions or the impact of using SDM for the health conditions in box 1 and (iii) provide a summary of outcomes from included studies. No restrictions will be applied to year or language of publication. This screening process will be conducted by one author with input from a second author. Potentially eligible records known to the international executive group will also be reviewed for eligibility and included if they provide new information (ie, report additional outcomes not identified through previous data sources).

Data extraction will be shared between two authors who will undertake line-by-line coding of included full-text articles. Every outcome and/or outcome domain will be extracted verbatim into a standard form (eg, Excel), alongside information about the source document (eg, first author). Extracted outcomes will be added to the long list.

#### Qualitative interviews

Interviews will elicit insights into further outcomes to be added to the long list as well as perspectives on the wording of outcomes and potential outcome domains.

Semi-structured, one-to-one interviews will be conducted with a diverse range of relevant international interest holders. Sampling will be purposive and will seek maximum variation by geography (ie, to ensure inclusivity across all continents), experience/expertise (ie, to ensure inclusivity across the COS healthcare conditions) and personal characteristics (ie, to ensure all key interest holder groups are represented).

A target sample of at least 20 participants will be recruited. This is comparable to other COS development studies^38–40^ with similarly broader scope and deemed of a minimum size to achieve ‘information power’.^41 42^ Relevant principles of information power will be considered when assessing final sample size requirements. For example, the aim of Stage 1 of this study is relatively focused, seeking to identify outcomes relevant to SDM rather than to explore experiences in-depth (item: study aim). Further, the purposive sampling strategy will ensure high sample specificity by recruiting interest holders with direct expertise or experience in SDM (item: sample specificity). Finally, this COS study has an existing empirical foundation provided by prior work undertaken to develop the rheuCOS-SDMi (item: established theory). These factors indicate that a minimum sample size of 20 participants is to be sufficient. A final sample size, however, will be determined iteratively through parallel data collection and analysis.

Patients and members of the public will be approached through existing patient and public involvement networks linked to members of the research team, by sending an advert via email to individuals who have previously expressed interest in this field of research. Experts in SDM will be approached similarly, by email, using the networks of the International Shared Decision-Making Society (ISDM). Snowballing will then be used to target clinicians and academics linked to country or regional leads for SDM research.

Interviews will be conducted in English, and online, by one researcher (JEB—an academic primary care physician and clinical lecturer with methodological expertise that includes qualitative research). Written consent will be obtained and verbally confirmed at the outset of interviews and participants will be given the opportunity to ask questions before providing their consent. Transcription software will be utilised, supplemented by audio recordings to check and correct transcripts. Any video-recorded data will be destroyed with only audio data retained. Transcripts will be pseudo-anonymised before analysis.

A topic guide has been developed to match the objectives of the study, with an introductory question to ask participants about what SDM means to them and any previous experience or expertise with SDM. The topic guide will be piloted and amended as necessary during the first three interviews.

A pragmatic constant comparative approach^43^ will be employed for the thematic analysis of interview transcripts. Coding will be carried out using NVivo software (V.14.23.1). Two researchers (JEB, and TN—a resident doctor with experience in qualitative methods and thematic analysis) will independently code a subset, compare, and if agreement is reached coding will continue by a single researcher. Coding will be both deductive and inductive. Deductive coding will follow the broad themes contained within the semi-structured topic guide. Inductive coding will capture new themes and theory not previously considered by the research team. Insights from the interviews will be used to add additional outcomes to the long list, not previously identified in other data sources.

#### Finalising the long list

Extracted outcomes/domains that were added to the long list will initially be mapped, where possible, onto domains identified in the rheuCOS-SDMi. Outcomes that do not align with existing rheuCOS-SDMi domains will be reviewed and grouped into further higher-order outcome domains (guided by the executive steering group and the PPAG) to be presented separately during subsequent stages. The process of grouping outcomes will be guided by existing frameworks, including the Outcome Measures in Rheumatoid Arthritis Clinical Trials (OMERACT) filter 2.1^44^ and the COMET outcome taxonomy,^45^ to create a long list of candidate outcome domains. Descriptions of outcome domains will draw on consensus-definitions agreed in the rheuCOS-SDMi or will be grounded in systematic review evidence, wherever possible. The long list will undergo at least two rounds of review by the executive group and the PPAG and iterative refinement. Members will be asked to independently review a draft of the long list to assess its overall comprehensibility and its comprehensiveness, structure and relevance to the new context. Written comments will be collected and separate meetings with executive group and PPAG members will also be organised to provide an opportunity to discuss and further elaborate on their feedback. The study delivery team (KT-A, AGKM, JEB, CH, MS, SL, TN) will collate and synthesise all feedback, and revisions will be made in response to identified issues. Attention will be given to refining wording (eg, outcome domain labels and explanations), resolving ambiguities, and ensuring that outcome domains are understandable and accessible for all interest holder groups. Further rounds of comments and revisions will be sought as appropriate to ensure all feedback has been appropriately addressed until a final long list is agreed to be taken forward to step 2.

### Step 2: Rating of the essentiality of outcome domains

A two-round international online Delphi will be used to agree on a shorter list of outcomes and outcome domains.

#### Delphi survey development

All outcome domains will be operationalised into an online Delphi questionnaire. Draft surveys will be piloted by members of the PPAG and selected individuals from the executive group and iteratively refined. This involves asking individuals to complete the full survey independently and provide written feedback to the study delivery team about the usability and face validity (ie, clarity and relevance) of the survey instructions and response scales, with identified issues addressed where appropriate.

The questionnaires will be hosted online using appropriate software (eg, REDCap). Questionnaires will be developed in English. The executive group considered multi-lingual COS development; however, a single language option was preferred to retain conceptual consistency during the survey development and because of concerns about feasibility within the resources available for the study.

#### Data collection

Participants will be invited to complete two consecutive rounds of the survey. In Round 1, participants are asked to provide sociodemographic details to determine the relevant panel group. During both rounds, participants will be asked to rate the importance of assessing each outcome domain using a Likert scale based on the Grading of Recommendations, Assessment, Development and Evaluations (GRADE) scale.^46^ This means scoring will be undertaken using a 9-point Likert scale with 1–3 labelled ‘not essential’, 4–6 labelled ‘important but not essential’ and 7–9 labelled ‘absolutely essential’.^46^ There will also be an ‘Unable to answer’ option for each question with the ability for the participant to enter free text to explain why they were not able to score the item. A single ‘free text’ response option at the end of the survey will be included to allow participants to suggest any additional domains. Participants will be informed that they should not base their responses to the survey on whether a validated measure is already available for a particular outcome domain. Outcome domains will be worded neutrally, in a way that can be interpreted bidirectionally without influence on the participant, that is, ‘patient trust’ as opposed to ‘more patient trust’ or ‘less patient trust’.

New potential outcome domains, identified by at least 10% of respondents through the free text response, will be included in subsequent Delphi rounds if deemed within scope. Only participants who complete Round 1 will be invited to Round 2. All questions will be mandatory to minimise missing data. Questions presented in Round 2 will be accompanied by feedback from Round 1 responses and participants will be asked to reconsider their response in light of this feedback. Feedback will consist of the average score for each question for each participant panel (ie, median score per outcome domain/question for each sub-group) and the individual participant’s own previous rating. All items will be retained in full between survey rounds 1 and 2. All items still retained after Round 2 of the Delphi survey will be taken forward to the consensus meeting where the final COS will be agreed.^32^ A third Delphi round will be considered if necessary.

#### Participant recruitment

The Delphi process will be undertaken inclusive of all interest holders relevant to this project. Participants will be recruited through adverts disseminated by established international SDM societies and OMERACT networks. Those approached will not be offered any financial incentive to participate. Participants will be purposively approached to ensure variation in key characteristics including sex, geographical location, ethnicity, age, healthcare experience or professional/clinical expertise (depending on the interest holder group). Participant characteristics will be reported using the PROGRESS+framework.^47^ Round 1 Delphi survey participants will be asked to confidentially provide their email address which will be used to facilitate recruitment for Round 2 Delphi survey. A direct email will be sent which will include an embedded link to the second round. A minimum of 150 participants per panel will be sought, in line with previous COS development studies.^32^ There will be no maximum sample size, and a weighted percentage will be considered should participant numbers be uneven across the different panels (eg patient/carer vs professionals).^32^

#### Analysis

Participant data will be separated into at least two Delphi panels depending on which interest holder group they represent. As a minimum, participants may be divided into patients, carers and family members (panel 1) and professionals including clinicians and SDM researchers (panel 2), to be able to assess differences in priorities and to avoid overrepresentation of a single group.

Participant response scores for the Delphi process will be analysed using descriptive statistics, adhering to the GRADE criteria.^46^ Analyses will be conducted separately for each panel and average scores calculated (ie, the median score of each panel per outcome domain/question). Results for each panel will be presented visually as feedback in Delphi Round 2. Final Delphi survey scores will again be subject to descriptive analyses with any changes in participant scores between rounds examined.

Response statistics and participant characteristics will be examined using descriptive statistics and presented in tabulated format by panel group. Attrition between Delphi survey rounds will be examined for potential bias in the different participant panels, comparing scores between participants who have completed only one Delphi survey round versus participants who have completed both Delphi survey rounds.

All analyses will be conducted with the help of a statistical software package (eg, Stata).

Recommended definitions of consensus will be followed during all rounds of the Delphi survey (see box 4).^36^

Box 4 **Consensus criteria**

- Consensus IN is defined as >70% of the total participants rating the outcome domain as 7–9 (‘absolutely essential’) and<15% rating the outcome domain as 1–3 (‘not essential’). This means the outcome domain will be taken forward to the consensus process.
- Consensus OUT is defined as >70% of participants rating the outcome domain as 1–3 (‘not essential’) and<15% rating the outcome domain as 7–9 (‘absolutely essential’). This means the outcome domain will not be taken forward to the consensus meeting.
- All other combinations of response results are defined as NO CONSENSUS.

### Step 3: Consensus on the final COS

Multiple international online consensus meetings will be conducted to discuss the results of the Delphi and, informed by the Delphi, to agree on a final COS-SDMi. This qualitative element provides an opportunity for experts in SDM to discuss and contextualise the quantitative Delphi findings. This approach also enables exploration of differing perspectives across interest holder groups, supports clarification and refinement of outcome domains, and facilitates transparent decision-making regarding inclusion, exclusion, or consolidation of outcomes.

The number of meetings will be determined in discussion with the executive group, considering the geographic representations and involvement of diverse interest holders. For example, it is anticipated that three meetings will be held to accommodate time zones in North and Latin America, Europe and Africa, Australia and Asia.

Recruitment for the consensus meetings will be targeted and facilitated through international SDM societies (eg, ISDM) and OMERACT networks, and key informant approaches. It is anticipated that no more than 25 participants, with fair representation of the interest holder groups listed above, will participate in each consensus meeting. A secure video conference platform will be used to host the meetings.

At each meeting, Delphi results will be presented by an independent chairperson with an understanding of SDM interventions. The Chair will lead structured discussions about the proposed inclusion/exclusion of domains from the COS, based on the consensus definitions. This will include discussions about combining outcomes depending on similarity. Any divergence in consensus between the two panel groups will be discussed. Interactive, anonymous voting will be used to gain consensus on proposed domains that achieve ‘NO CONSENSUS’ after the Delphi rounds. Those domains achieving ‘IN’ or ‘OUT’ status after the Delphi will simply be presented as such, and voting on these will only take place if there is sufficient objection to the status of these domains. Should a large number of outcome domains achieve ‘IN’ status after the Delphi, discussions will explore consolidating them into higher-order outcome domains.

The results of the multiple online consensus meetings will be synthesised, reported and considered at a hybrid (both online and in-person) symposium at an international conference (eg, the bi-annual ISDM conference) attended by patient and professional interest holders from diverse backgrounds. This process will result in the reporting of the final COS-SDMi.

Future work will develop a core measurement set to identify instruments to assess agreed core outcomes and finalise outcome domain definitions.

## Ethics and dissemination

Research ethics approval has been granted in the UK by the University of Bristol Faculty Ethics Committee (ref: 7741) and the University of Exeter Health and Community Sciences Ethics Committee (ref: 8207624). Written informed consent will be obtained digitally from all participants in the qualitative interviews, the Delphi surveys and the consensus meetings.

The final COS will be disseminated by presentation at international SDM conferences and briefing notes to key organisations. Results of this study will be published in a peer-reviewed journal. Tailored to the needs of a wide, diverse audience, the delivery group will consider the production of plain language summaries, infographics, and/or translated materials, as appropriate. Further dissemination is planned through the patient/public advisory group, ISDM, OMERACT, and executive group channels, to publicise the COS to patient groups, funders, journal editors, regulatory bodies and HTA boards internationally.

## References

[1] Charles C, Gafni A, Whelan T (1997). Shared decision-making in the medical encounter: What does it mean? (or it takes at least two to tango). *Social Science & Medicine*.

[2] Härter M, Moumjid N, Cornuz J (2017). Shared decision making in 2017: International accomplishments in policy, research and implementation. Z Evid Fortbild Qual Gesundhwes.

[3] Department of Health and Social Care (2021). The NHS constitution for England. https://www.gov.uk/government/publications/the-nhs-constitution-for-england/the-nhs-constitution-for-england#introduction-to-the-nhs-constitution.

[4] Royal College of Surgeons (2013). Good surgical practice.

[5] General Medical Council (2013). Good medical practice.

[6] National Institute for Health and Care Excellence (2021). Shard decision making [NG197]. Evidence reviews 2021.

[7] Department of Health (2012). Liberating the NHS: No decision about me without me - Government response. Dep Heal.

[8] Légaré F, Adekpedjou R, Stacey D (2018). Interventions for increasing the use of shared decision making by healthcare professionals. Cochrane Database Syst Rev.

[9] Hawe P (2015). Lessons from complex interventions to improve health. Annu Rev Public Health.

[10] Sarri G, Freitag A, Szegvari B (2021). The Role of Patient Experience in the Value Assessment of Complex Technologies – Do HTA Bodies Need to Reconsider How Value is Assessed?. *Health Policy*.

[11] Jen M-H, Mt-Isa S, Chu H (2026). The Current Landscape of HTA Framework and Key Challenges. Stat Biopharm Res.

[12] Elwyn G, Lloyd A, May C (2014). Collaborative deliberation: a model for patient care. Patient Educ Couns.

[13] Elwyn G, Frosch DL, Kobrin S (2016). Implementing shared decision-making: Consider all the consequences. Implement Sci.

[14] Hughes TM, Merath K, Chen Q (2018). Association of shared decision-making on patient-reported health outcomes and healthcare utilization. Am J Surg.

[15] Arterburn D, Wellman R, Westbrook E (2012). Introducing Decision Aids At Group Health Was Linked To Sharply Lower Hip And Knee Surgery Rates And Costs. Health Aff.

[16] Heisler M, Bouknight RR, Hayward RA (2002). The relative importance of physician communication, participatory decision making, and patient understanding in diabetes self-management. J Gen Intern Med.

[17] Pieterse AH, Kunneman M, van den Hout WB (2019). Patient explicit consideration of tradeoffs in decision making about rectal cancer treatment: benefits for decision process and quality of life. Acta Oncol (Madr).

[18] Lantz PM, Janz NK, Fagerlin A (2005). Satisfaction with surgery outcomes and the decision process in a population-based sample of women with breast cancer. Health Serv Res.

[19] Langseth MS, Shepherd E, Thomson R (2012). Quality of decision making is related to decision outcome for patients with cardiac arrhythmia. Patient Educ Couns.

[20] Stacey D, Bennett CL, Barry MJ (2011). Decision aids for people facing health treatment or screening decisions. *Cochrane Database Syst Rev*.

[21] Cuypers M, Lamers RED, Kil PJM (2018). Impact of a web-based prostate cancer treatment decision aid on patient-reported decision process parameters: results from the Prostate Cancer Patient Centered Care trial. Support Care Cancer.

[22] Burton D, Blundell N, Jones M (2010). Shared decision-making in cardiology: do patients want it and do doctors provide it?. Patient Educ Couns.

[23] Ananian P, Houvenaeghel G, Protière C (2004). Determinants of patients’ choice of reconstruction with mastectomy for primary breast cancer. Ann Surg Oncol.

[24] Wallen GR, Brooks AT (2012). To Tell or Not to Tell: Shared Decision Making, CAM Use and Disclosure Among Underserved Patients with Rheumatic Diseases. Integr Med Insights.

[25] Schoenthaler AM, Schwartz BS, Wood C (2012). Patient and physician factors associated with adherence to diabetes medications. Diabetes Educ.

[26] Rehfuess EA, Booth A, Brereton L (2018). Towards a taxonomy of logic models in systematic reviews and health technology assessments: A priori, staged, and iterative approaches. Res Synth Methods.

[27] McCulloch P, Nagendran M, Campbell WB (2013). Strategies to reduce variation in the use of surgery. Lancet.

[28] Reames BN, Shubeck SP, Birkmeyer JD (2014). Strategies for reducing regional variation in the use of surgery: a systematic review. Ann Surg.

[29] Leinweber KA, Columbo JA, Kang R (2019). A Review of Decision Aids for Patients Considering More Than One Type of Invasive Treatment. J Surg Res.

[30] Kunneman M, Henselmans I, Gärtner FR (2019). Do Shared Decision-Making Measures Reflect Key Elements of Shared Decision Making? A Content Review of Coding Schemes. Med Decis Mak.

[31] Toupin-April K, Décary S, de Wit M (2021). Endorsement of the OMERACT core domain set for shared decision making interventions in rheumatology trials: Results from a multi-stepped consensus-building approach. Semin Arthritis Rheum.

[32] Kirkham JJ, Davis K, Altman DG (2017). Core Outcome Set-STAndards for Development: The COS-STAD recommendations. PLoS Med.

[33] National Institute for Health and Care Excellence (2021). Shared decision making [A] Evidence review for effectiveness of approaches and activities to increase engagement in shared decision making and the barriers and facilitators to engagement.

[34] National Institute for Health and Care Excellence (2021). Shared decision making [B] Evidence review for interventions to support effective shared decision making.

[35] Mcleroy KR, Bibeau D, Steckler A (1988). An Ecological Perspective on Health Promotion Programs. Heal Educ Behav.

[36] Williamson PR, Altman DG, Bagley H (2017). The COMET Handbook: version 1.0. Trials.

[37] Kirkham JJ, Gorst S, Altman DG (2016). Core Outcome Set-STAndards for Reporting: The COS-STAR Statement. PLoS Med.

[38] Alkhaffaf B, Metryka A, Blazeby JM (2021). Core outcome set for surgical trials in gastric cancer (GASTROS study): international patient and healthcare professional consensus. Br J Surg.

[39] Lee SI, Hanley S, Vowles Z (2023). The development of a core outcome set for studies of pregnant women with multimorbidity. BMC Med.

[40] Lee N, Smith C, Bailey R (2025). Core Outcome Set development for LEPtospirosis trials (COS-LEP): a study protocol to develop a core outcome set for the evaluation of clinical therapeutic interventions for human leptospirosis. Trials.

[41] Malterud K, Siersma VD, Guassora AD (2016). Sample Size in Qualitative Interview Studies: Guided by Information Power. Qual Health Res.

[42] Green J, Thorogood N (2025). Qualitative methods for health research.

[43] Maykut P, Morehouse R (2021). Qualitative Data Analysis: Using the Constant Comparative Method. Beginning Qualitative Research.

[44] Boers M, Beaton DE, Shea BJ (2019). OMERACT Filter 2.1: Elaboration of the Conceptual Framework for Outcome Measurement in Health Intervention Studies. J Rheumatol.

[45] Dodd S, Clarke M, Becker L (2018). A taxonomy has been developed for outcomes in medical research to help improve knowledge discovery. J Clin Epidemiol.

[46] Guyatt GH, Oxman AD, Kunz R (2011). GRADE guidelines: 2. Framing the question and deciding on important outcomes. J Clin Epidemiol.

[47] O’Neill J, Tabish H, Welch V (2014). Applying an equity lens to interventions: using PROGRESS ensures consideration of socially stratifying factors to illuminate inequities in health. J Clin Epidemiol.

[48] Jull J, Köpke S, Smith M (2021). Decision coaching for people making healthcare decisions. Cochrane Database Syst Rev.

[49] Wehking F, Debrouwere M, Danner M (2024). Impact of shared decision making on healthcare in recent literature: a scoping review using a novel taxonomy. J Public Health (Berl).

[50] Gültzow T, Zijlstra DN, Bolman C (2021). Decision aids to facilitate decision making around behavior change in the field of health promotion: A scoping review. Patient Educ Couns.

[51] Ara R, Brazier J, Sculpher M (2024). A framework for conducting economic evaluations when using patient decision aids in health care decision making. Published Online First.

