# Supplementary material for: Development of a core outcome set for the evaluation of shared decision-making interventions in healthcare: a study protocol
Source: BMJ Open. 2026 Sep 15;16(9):e116623. doi: 10.1136/bmjopen-2026-116623 (PMC13583826; doi:10.1136/bmjopen-2026-116623)
Supplement: Supplementary file 1 [file bmjopen-2026-116623.reviewer_comments.pdf]

PEER REVIEW HISTORY

BMJ Open publishes all reviews undertaken for accepted manuscripts. Reviewers are asked to complete a checklist review form and are provided with free text boxes to elaborate on their assessment. These free text comments are reproduced below.

ARTICLE DETAILS

Title (Provisional)

Development of a core outcome set for the evaluation of shared decision-making interventions in healthcare: a study protocol

Authors

Hoffmann, Christin; Butterworth, Joanne E.; Naye, Florian; Smith, Maureen; Nyamapfene, Taona; Lawday, Samuel; Avery, Kerry; Bekker, Hilary; Bravo, Paulina; Décary, Simon; Edwards, Adrian; Elwyn, Glyn; Engelhardt, Ellen G.; Franco, Juan Victor Ariel; Garvelink, Mirjam M; Giguere, Anik MC; Härter, Martin; Hoffmann, Tammy; Kienlin, Simone; McCaffery, Kirsten; Noordman, Janneke; Olling, Karina; Perestelo-Pérez, Lilisbeth; Pieterse, Arwen H.; Scheibler, Fülöp; Sepucha, Karen; Ubbink, Dirk; Valentine, KD; Volk, Robert J; Wehking, Felix; Yoo, Sang Ho; Bulbeck, Helen; Cole, Amy C; de Wit, Maarten; Finderup, Jeanette; Geary, Katie; Gunn, Christine M.; Hou, Wen-Hsuan; Housten, Ashley J.; Kim, Min Ji; Norma, Marti; Pacheco-Brousseau, Lissa; Snede, Lisbeth; Tian, Cindy; McNair, Angus GK; Toupin-April, Karine

---

VERSION 1 - REVIEW

**Reviewer**	**1**
**Name**	**Bui, Michael**
**Affiliation**	**University of Twente, Health Technology and Services Research**
**Date**	**24-Mar-2026**
**COI**	**None**

Thank you for giving me the opportunity to review this manuscript. This protocol outlines an ambitious and clinically relevant effort to develop a core outcome set for shared decision-making applicable across health conditions. The development of this core outcome set could lead to greater standardisation in reporting across studies, and thereby facilitate meta-analyses to strengthen our understanding on the impact of shared decision-making. However, I foresee several issues that should be addressed before the manuscript is suitable for publication. Especially since this is a research protocol paper, I believe that several methodological considerations warrant more transparent justifications.

Major concerns:

1. Introduction
    a. In lines 22-26, the authors argue that high-quality health technology assessment (HTA) requires an understanding of how interventions facilitate the adoption and implementation of SDM impact clinical health services outcomes beyond process and patient experience. I do not quite understand what is meant by this. In particular, I don't understand why SDM is relevant to HTA. Upon checking the reference (9) that was used to substantiate this claim, I could not find any hits for shared decision-making in the paper. Furthermore, in a recent eDelphi study on patient-centred HTA, SDM was not mentioned in the consensus recommendations either (DOI: 10.1016/j.jval.2025.06.014). Please clarify the message you are trying to convey here, and verify whether your reference is used appropriately. Clarification of this statement is also warranted in your abstract, as you also use it there.
    b. The authors cited reference (31) to illustrate that the use of decision aids significantly reduced rates of prostatectomy, hip/knee replacement, and hysterectomy. But a reduction in these procedures does not directly imply that patients experienced better outcomes as a result of SDM. Could you elaborate on the clinical relevance of the observation you made here?
2. Methods and analysis
    a. In the section titled: "Review of existing literature", the review procedure lacks specificity. Various items required in reporting guidelines such as PRISMA are not present, which limit the reproducibility of the study. I recommend to authors to report adhere to the PRISMA guidelines, to clarify the following in particular:
        i. The inclusion and exclusion criteria are not described concretely. "Review papers also have to have a broad scope so that results can be generalisable" is too vague. When is a scope broad? Is that the case when the review focuses on more than one disease? Or when the review focuses on a whole healthcare domain, rather than a specific classes of diseases? Without clear inclusion and exclusion criteria, it is also difficult for me to judge as a reviewer whether your initial search would be sufficiently comprehensive for the development of your core outcome set.
        ii. The search strategy is not described transparently. The authors mention "additional data sources", but it is unclear what the primary data sources are, i.e., which data bases are searched. Additionally, it is unclear which key concepts will be used to inform the search strategy, and whether any filters / restrictions will be applied, e.g., based on language of publication or year of publication.
        iii. For the data extraction, it is not clear how many authors are involved in the data extraction. Additionally, it is unclear whether you will base your extractions on the primary studies included in the reviews you identified, or whether you only extract information from the reviews themselves.
    b. There are several unclarities In the section titled: "Primary qualitative interviews".
        i. First, the authors argue that a target sample of 20 participants is satisfactory to ensure sufficient information power, based on studies using similar methodologies. However, this reasoning lacks transparency. To justify the sample size, the authors have cited an article by Malterud et al. (2016). This article states that sample size determination depends on: "(a) the aim of the study, (b) sample specificity, (c) use of established theory, (d) quality of dialogue, and (e) analysis strategy". None of these five aspects are explicitly

addressed in the current protocol. In particular, I would expect that judgements based on sample specificity would demand a larger sample size. The authors have defined a very broad scope for the intended COS-SDM, e.g., applicability across all health conditions and populations who are involved in healthcare decision-making. Given this scope, I'm not sure whether all relevant experiences can be represented by 20 participants. I'd like to see a more transparent justification for the targeted sample size.

   ii. Second, the authors state that sampling will be done by "personal characteristics". Please be more explicit about what this exactly entails.

c. I have several remarks about the section titled: "Step 2: Prioritisation of core domains".

   i. First, I'm not sure whether the term 'prioritisation' is appropriate here. You propose to assess the essentiality of the items using a Likert scale. But strictly speaking, this rating instrument does not explicitly ask respondents to prioritise outcomes. You proposed a 9-point Likert scale, and will presumably have more than 9 core outcomes to rate. By definition, your participants will then not be able to rank, i.e., prioritise, all core outcomes with such a restricted scale. A more appropriate section title would be something along the lines of "Step 2: Rating the essentiality of core domains".

   ii. Second, the Delphi round incorporates a rating exercise to determine the essentiality of the outcomes, but does not inquire on the clarity of the outcome descriptions. As you are developing a new core outcome set, I'd argue that establishing content validity is important, for which clarity assessments are currently missing. While the authors propose to involve the PPAG in assessing the clarity and comprehensibility of the survey, this does not guarantee that future users of your core outcome set (e.g., your survey participants), will also deem the outcome descriptions clear. In my experience with Delphi studies, at least some degree of rewording of the outcomes is likely required to resolve unclarities. Hence, I do not think that merely focusing on essentiality in your survey is sufficient. Please address how you plan to accommodate for ensuring your core outcome set description is clear.

   iii. Third, the authors state that the face validity will be examined by members of the PPAG. However, it is not clear which formal procedures are undertaken to establish face validity.

d. Several clarifications are required in the section titled "Step 3: Reaching consensus".

   i. First, I find the following methodological decision somewhat arbitrary: "Definitions may be revised for Round 2 analyses, in case stricter criteria (e.g., higher percentage thresholds) are necessary because of a high number of participants rating outcome domains as 'absolutely essential' [7-9]." To me, this sounds like a prioritisation problem: amongst the outcomes that are all deemed essential to retain according to your consensus definition, you want to select a subset to ensure that the COS size remains practically manageable. I'm not sure whether modifying your percentage threshold is a valid approach towards prioritisation. Especially since you never formally ask your respondents to prioritise outcomes when you use a Likert scale as your measurement instrument. Methodologically, I think you first need to obtain

expert input on what the maximum permissible COS size would be. Subsequently, you would need an elicitation method that actually measures the importance of outcomes, from which you can derive a ranking, such as best-worst scaling case 1. Then from this prioritisation exercise, you could select the top-N most important outcomes for your COS, where N is the maximum permissible COS size.

ii. Second, I'm somewhat confused about the proposed approach towards establishing consensus. On the one hand, you are using a two-round Delphi study with a targeted sample size of 150 respondents to determine consensus based on essentiality ratings. But for the final consensus, you propose a nominal group technique with at most 25 participants to determine the final core outcome selection. What is the methodological rationale for this approach, rather than conducting a three-round Delphi study with consistent engagement of all 150 participants?

VERSION 1 - AUTHOR RESPONSE

Reviewer: 1

Mr. Michael Bui, University of Twente

Comments to the Author:

Thank you for giving me the opportunity to review this manuscript. This protocol outlines an ambitious and clinically relevant effort to develop a core outcome set for shared decision-making applicable across health conditions. The development of this core outcome set could lead to greater standardisation in reporting across studies, and thereby facilitate meta-analyses to strengthen our understanding on the impact of shared decision-making. However, I foresee several issues that should be addressed before the manuscript is suitable for publication. Especially since this is a research protocol paper, I believe that several methodological considerations warrant more transparent justifications.

Response

We thank the reviewer for their thorough review of our manuscript and helpful suggestions to improve the protocol of our study. We have responded to each comment and have highlighted the relevant changes made.

Comment 2.1

Major concerns:

1. Introduction

a. In lines 22-26, the authors argue that high-quality health technology assessment (HTA) requires an understanding of how interventions facilitate the adoption and implementation of SDM impact clinical health services outcomes beyond process and

patient experience. I do not quite understand what is meant by this. In particular, I don't understand why SDM is relevant to HTA. Upon checking the reference (9) that was used to substantiate this claim, I could not find any hits for shared decision-making in the paper. Furthermore, in a recent eDelphi study on patient-centred HTA, SDM was not mentioned in the consensus recommendations either (DOI: 10.1016/j.jval.2025.06.014). Please clarify the message you are trying to convey here, and verify whether your reference is used appropriately. Clarification of this statement is also warranted in your abstract, as you also use it there.

Response

Thank you for raising this point. We agree that this paragraph required further clarification and have implemented changes to restructure it and further clarify the relevance of HTA by stating:

"Health technology assessment (HTA) is the process to evaluate the impact of health interventions and inform evidence-based decision making in healthcare systems by assessing value beyond efficacy alone (11). High-quality HTA therefore requires an understanding of how interventions designed to facilitate the adoption and implementation of SDM influence clinical and health service outcomes, extending beyond process and patient experience. "

Health technology assessment (HTA) refers to the commonly used, systematic way of evaluating new medical technologies to inform healthcare decisions. Its purpose is to inform decision-making in healthcare. A health technology is generally used as a generic term to mean a range of interventions, including drugs, medical devices, treatments/procedures or health programmes and service models. It is the gold standard way in many modern healthcare systems to determine clinical and cost effectiveness of a new intervention, such as those aiming to improve shared decision making (SDM). We would not expect SDM interventions to be explicitly referenced in generic HTA guidelines as these are designed to be applicable to this broad range of interventions. However, we intend for this work to improve the visibility of SDM interventions as healthcare interventions and as such, their relevance to HTA.

Comment 2.2

b. The authors cited reference (31) to illustrate that the use of decision aids significantly reduced rates of prostatectomy, hip/knee replacement, and hysterectomy. But a reduction in these procedures does not directly imply that patients experienced better outcomes as a result of SDM. Could you elaborate on the clinical relevance of the observation you made here?

Response

Thank you for the opportunity to elaborate on this example. We have explained the clinical relevance. The amended paragraph now reads:

"Three demonstrated statistically significant reductions in rates of prostatectomy, hip/knee replacement, and hysterectomy. This suggests that decision aids can support patients in making informed, values-based treatment decisions, with the observed reduction in surgery rates likely reflecting that some patients, when better informed about their options, choose more conservative treatments that align with their preferences [29]. This may have implications for healthcare resource use and costs through a lower number of surgical procedures performed. There may also be downstream implication for procedure-related risks and consequences through avoiding complications and healthcare burden and costs for patients."

Comment 2.3

2. Methods and analysis

a. In the section titled: "Review of existing literature", the review procedure lacks specificity. Various items required in reporting guidelines such as PRISMA are not present, which limit the reproducibility of the study. I recommend to authors to report adhere to the PRISMA guidelines, to clarify the following in particular:

i. The inclusion and exclusion criteria are not described concretely. "Review papers also have to have a broad scope so that results can be generalisable" is too vague. When is a scope broad? Is that the case when the review focuses on more than one disease? Or when the review focuses on a whole healthcare domain, rather than a specific classes of diseases? Without clear inclusion and exclusion criteria, it is also difficult for me to judge as a reviewer whether your initial search would be sufficiently comprehensive for the development of your core outcome set.

ii. The search strategy is not described transparently. The authors mention "additional data sources", but it is unclear what the primary data sources are, i.e., which data bases are searched. Additionally, it is unclear which key concepts will be used to inform the search strategy, and whether any filters / restrictions will be applied, e.g., based on language of publication or year of publication.

iii. For the data extraction, it is not clear how many authors are involved in the data extraction. Additionally, it is unclear whether you will base your extractions on the primary studies included in the reviews you identified, or whether you only extract information from the reviews themselves.

Response

Thank you for raising these points and we agree that further clarification of our methods to produce the long list was needed. We have now clearly outlined our 3 different types of data sources throughout the manuscript which were: 1) the existing SDM COS developed in the context of Rheumatology (rheuCOS-SDMi), 2) targeted searches of existing review studies, and 3) qualitative interviews. Major changes were implemented across the Methods section (in particular, pp. 13-15) to improve clarity and add detail, including the exact references of studies included for data source 1), the eligibility criteria and extraction methods for data source 2).

Importantly, we have clarified that the second stage involved targeted searches, rather than a systematic search, where methodological steps and reporting typical for systematic reviews (e.g. search strategy) do not apply. A PRISMA diagram will be considered for inclusion in the publication once the search results are known. We deemed a targeted search an appropriate way to identify relevant outcomes with sufficiently high confidence because of the robust and comprehensive methods used during the development of the rheuCOS-SDMi and the existing Cochrane review of SDM interventions.

Comment 2.4

b. There are several unclarities In the section titled: “Primary qualitative interviews”.

i. First, the authors argue that a target sample of 20 participants is satisfactory to ensure sufficient information power, based on studies using similar methodologies. However, this reasoning lacks transparency. To justify the sample size, the authors have cited an article by Malterud et al. (2016). This article states that sample size determination depends on: “(a) the aim of the study, (b) sample specificity, (c) use of established theory, (d) quality of dialogue, and (e) analysis strategy”. None of these five aspects are explicitly addressed in the current protocol. In particular, I would expect that judgements based on sample specificity would demand a larger sample size. The authors have defined a very broad scope for the intended COS-SDM, e.g., applicability across all health conditions and populations who are involved in healthcare decision-making. Given this scope, I’m not sure whether all relevant experiences can be represented by 20 participants. I’d like to see a more transparent justification for the targeted sample size.

Response

Thank you for the opportunity to elaborate on the methods for our qualitative study component and add justifications for our sample size. We agree and have reworded to state that we aim to recruit **a minimum of 20** participants and have included

justification based on the principles outlined in Malterud's framework. The section now reads:

"A target sample of at least 20 participants will be recruited. This is comparable to other COS development studies [1–3] *with similarly broader scope and deemed of a minimum size to achieve 'information power'* [4,5]. *Relevant principles of information power will be considered when assessing final sample size requirements. For example, the aim of Stage 1 of this study is relatively focused, seeking to identify outcomes relevant to SDM rather than to explore experiences in-depth (item: study aim). Further, the purposive sampling strategy will ensure high sample specificity by recruiting interest holders with direct expertise or experience in SDM (item: sample specificity). Finally, this COS study has an existing empirical foundation provided by prior work undertaken to develop the rheuCOS-SDMi (item: established theory). These factors indicate that a minimum sample size of 20 participants is to be sufficient. A final sample size, however, will be determined iteratively through parallel data collection and analysis."*

Comment 2.5

ii. Second, the authors state that sampling will be done by "personal characteristics". Please be more explicit about what this exactly entails.

Response

Again, thank you for highlighting this. We have added further detail to this section which now reads:

"Sampling will be purposive and will seek maximum variation by geography (i.e., to ensure inclusivity across all continents), experience/expertise (i.e., to ensure inclusivity across the COS healthcare conditions), and personal characteristics (i.e., to ensure all key interest holder groups are represented)."

Comment 2.6

c. I have several remarks about the section titled: "Step 2: Prioritisation of core domains".

i. First, I'm not sure whether the term 'prioritisation' is appropriate here. You propose to assess the essentiality of the items using a Likert scale. But strictly speaking, this rating instrument does not explicitly ask respondents to prioritise outcomes. You proposed a 9-point Likert scale, and will presumably have more than 9 core outcomes to rate. By definition, your participants will then not be able to rank, i.e., prioritise, all core outcomes with such a restricted scale. A more appropriate section title would be

something along the lines of “Step 2: Rating the essentiality of core domains”.

Response

Thank you for this suggestion and we agree with the reviewer that using a rating instrument does not explicitly ask respondents to prioritise outcomes. We therefore modified the wording to “Step 2: Rating of the essentiality of outcome domains” and have carefully reviewed the manuscript to ensure this change is reflected throughout (e.g. Abstract).

Comment 2.7

ii. Second, the Delphi round incorporates a rating exercise to determine the essentiality of the outcomes, but does not inquire on the clarity of the outcome descriptions. As you are developing a new core outcome set, I’d argue that establishing content validity is important, for which clarity assessments are currently missing. While the authors propose to involve the PPAG in assessing the clarity and comprehensibility of the survey, this does not guarantee that future users of your core outcome set (e.g., your survey participants), will also deem the outcome descriptions clear. In my experience with Delphi studies, at least some degree of rewording of the outcomes is likely required to resolve unclarities. Hence, I do not think that merely focusing on essentiality in your survey is sufficient. Please address how you plan to accommodate for ensuring your core outcome set description is clear.

Response

Thank you for raising this important point. We agree that clarity and comprehensibility of core outcome domains are critical. We have considered the inclusion of questions that ask about the clarity for each outcome domain. However, due to a potentially large number of items, we did not want to increase participant burden. Instead, participants in the Delphi process will have the opportunity to provide free-text comments alongside their ratings, allowing them to add any views about clarity or comprehensibility. We have further elaborated on this in the relevant ‘Data extraction’ section (pp. 17-18).

We have taken several steps to maximise the Delphi content validity, including several rounds of multi-stakeholder input and pilot testing, alongside iterative refinements of the survey. Further, the candidate outcome domains in the Delphi will be informed by prior work, including domains defined in the rheuCOS-SDMi and existing high-quality systematic reviews of SDM interventions. This means that candidate outcomes are not entirely new and initial wording will be grounded in the established literature.

It is important to highlight too, that the formal development process for outcome domain descriptions is outside the scope of this study, in line with COMET guidance. This phase focusses on the identification and agreement of outcome domains for inclusion in the core outcome set. Detailed specification and standardisation of outcome definitions is typically undertaken in subsequent phase, which is the development of a core measurement set. We have now included a sentence at the end of the manuscript which reads:

"Future work will develop a core measurement set to identify instruments to assess agreed core outcomes and finalise outcome domain definitions."

Comment 2.8

iii. Third, the authors state that the face validity will be examined by members of the PPAG. However, it is not clear which formal procedures are undertaken to establish face validity.

Response

Thank you for highlighting this and we agree this was not clear. We have added further detail about the PPAG's and exec group members involvement in this process, which also addressed Comment 2.8.

"All outcome domains will be operationalised into an online Delphi questionnaire. Draft surveys will be piloted by members of the PPAG and selected individuals from the executive group and iteratively refined. This involves asking individuals to complete the full survey independently and provide written feedback to the study delivery team about the usability and face validity (i.e., clarity and relevance) of the survey instructions and response scales, with identified issues addressed where appropriate."

Comment 2.9

d. Several clarifications are required in the section titled "Step 3: Reaching consensus".
i. First, I find the following methodological decision somewhat arbitrary: "Definitions may be revised for Round 2 analyses, in case stricter criteria (e.g., higher percentage thresholds) are necessary because of a high number of participants rating outcome domains as 'absolutely essential' [7-9]." To me, this sounds like a prioritisation problem: amongst the outcomes that are all deemed essential to retain according to your consensus definition, you want to select a subset to ensure that the COS size remains practically manageable. I'm not sure whether modifying your percentage threshold is a valid approach towards prioritisation. Especially since you never formally ask your

respondents to prioritise outcomes when you use a Likert scale as your measurement instrument. Methodologically, I think you first need to obtain expert input on what the maximum permissible COS size would be. Subsequently, you would need an elicitation method that actually measures the importance of outcomes, from which you can derive a ranking, such as best-worst scaling case 1. Then from this prioritisation exercise, you could select the top-N most important outcomes for your COS, where N is the maximum permissible COS size.

Response

Thank you for your query and the opportunity to address this. We have again reviewed the literature and whilst other COS development studies [9–12] have indeed used adaptation of percentage thresholds to address issues of ceiling effects, we prefer to follow the reviewer's suggestion and the COMET handbook guidance [13] to clearly define the consensus criteria in our protocol. We have therefore removed any reference to potential modifications to the consensus thresholds. We have also now outlined our plans for addressing potentially high numbers of outcome domains to be brought forward into the consensus by adding to Step 3:

"Should a large number of outcome domains achieve 'IN' status after the Delphi, discussions will explore consolidating them into higher-order outcome domains."

We agree with the reviewer that the Delphi process and use of Likert-scale ratings is not generating a ranked ordering of outcome domains. However, the aim is not to develop a ranked list of final outcomes, but to provide an unordered set of important outcome domains as agreed by all interest holders. The final number of core outcomes is not pre-defined, but consensus on these involves a mixture of quantitative (Delphi) and qualitative (nominal group meetings) approaches to achieve agreement on a final core set that is endorsed by all key interest holders. We do not consider it appropriate to introduce alternative consensus methodologies at this stage, as it would represent a substantial deviation from established COMET Delphi methodology [6], which recommends the use of the specific Likert scales and endpoints, including the proposed analysis [14].

Comment 2.10

ii. Second, I'm somewhat confused about the proposed approach towards establishing consensus. On the one hand, you are using a two-round Delphi study with a targeted sample size of 150 respondents to determine consensus based on essentiality ratings. But for the final consensus, you propose a nominal group technique with at most 25 participants to determine the final core outcome selection. What is the methodological

rationale for this approach, rather than conducting a three-round Delphi study with consistent engagement of all 150 participants?

Response

Thank you for this query. We have now added a justification to the section "Step 3" to explain the purpose of consensus meeting. This reads:

"Multiple international online consensus meetings will be conducted to discuss the results of the Delphi and agree on a final core outcome set. This qualitative element provides an opportunity for experts in SDM to discuss and contextualise the quantitative Delphi findings. This approach also enables exploration of differing perspectives across interest holder groups, supports clarification and refinement of outcome domains, and facilitates transparent decision-making regarding inclusion, exclusion, or consolidation of outcomes."

This study follows COMET methodology for the development of core outcome sets, which is currently considered best-practice (gold standard). This includes a multi-step process including evidence reviews, Delphi and consensus meeting(s). Consensus meetings are a standard and common final step in COS development to ratify the Delphi results. It also provides a final opportunity for key interest holders to discuss in more depth any discrepancies and/or raise concerns about ratifying the final COS.

These methods are also replicated in a vast range of contexts and for a wide range of health conditions and situations. The evidence for the methodological underpinnings and justification for each step are explained in the COMET Handbook and COS development guidance [6,15]. For a comprehensive overview of studies applying this method, please see the annual systematic review updates by Gorst et al [16–18] and the COMET database (https://www.comet-initiative.org/) which provide a detailed synthesis and record of core outcome set development studies, respectively.

We have performed additional amendments to the manuscript, including wording and heading changes, to correct typos, improve clarity and specific, and address inconsistencies.

References

1 Alkhaffaf B, Metryka A, Blazeby JM, *et al.* Core outcome set for surgical trials in gastric cancer (GASTROS study): international patient and healthcare professional consensus. *Br J Surg*. 2021;108:1216–24. doi: 10.1093/BJS/ZNAB192

2 Lee SI, Hanley S, Vowles Z, *et al.* The development of a core outcome set for studies of pregnant women with multimorbidity. *BMC Med 2023 211*. 2023;21:314-. doi: 10.1186/S12916-023-03013-3

3 Lee N, Smith C, Bailey R, *et al.* Core Outcome Set development for LEPtospirosis trials (COS-LEP): a study protocol to develop a core outcome set for the

evaluation of clinical therapeutic interventions for human leptospirosis. *Trials 2025 261*. 2025;26:6-. doi: 10.1186/S13063-024-08713-6

4 Malterud K, Siersma VD, Guassora AD. Sample Size in Qualitative Interview Studies: Guided by Information Power. *Qual Health Res*. 2016;26:1753–60. doi: 10.1177/1049732315617444;WEBSITE:WEBSITE:SAGE;REQUESTEDJOURNAL:JOURNAL:QHRA;JOURNAL:JOURNAL:QHRA;WGROUP:STRING:PUBLICATION

5 Green J, Thorogood N. *Qualitative Methods for Health Research*. SAGE Publications Ltd 2025.

6 Williamson PR, Altman DG, Bagley H, *et al.* The COMET Handbook: Version 1.0. Trials. 2017;18:1–50.

7 Boers M, Beaton DE, Shea BJ, *et al.* OMERACT filter 2.1: Elaboration of the conceptual framework for outcome measurement in health intervention studies. *J Rheumatol*. 2019;46:1021–7. doi: 10.3899/JRHEUM.181096

8 Dodd S, Clarke M, Becker L, *et al.* A taxonomy has been developed for outcomes in medical research to help improve knowledge discovery. *J Clin Epidemiol*. 2018;96:84–92. doi: 10.1016/j.jclinepi.2017.12.020

9 Avery KNL, Wilson N, MacEfield R, *et al.* A Core Outcome Set for Seamless, Standardized Evaluation of Innovative Surgical Procedures and Devices (COHESIVE): A Patient and Professional Stakeholder Consensus Study. *Ann Surg*. 2023;277:238–45. doi: 10.1097/SLA.0000000000004975

10 Korte EWH, van den Akker PC, Kiritsi D, *et al.* Core outcome domain sets for clinical trials in epidermolysis bullosa — a COSEB protocol to achieve consensus on “what” to measure. *Trials*. 2025;26:399. doi: 10.1186/S13063-025-09052-W

11 McNair AGK, Whistance RN, Forsythe RO, *et al.* Core Outcomes for Colorectal Cancer Surgery: A Consensus Study. *PLOS Med*. 2016;13:e1002071. doi: 10.1371/JOURNAL.PMED.1002071

12 Davies TW, van Gassel RJJ, van de Poll M, *et al.* Core outcome measures for clinical effectiveness trials of nutritional and metabolic interventions in critical illness: an international modified Delphi consensus study evaluation (CONCISE). *Crit Care 2022 261*. 2022;26:240-. doi: 10.1186/S13054-022-04113-X

13 Sinha IP, Gallagher R, Williamson PR, *et al.* Development of a core outcome set for clinical trials in childhood asthma: a survey of clinicians, parents, and young people. *Trials 2012 131*. 2012;13:1–9. doi: 10.1186/1745-6215-13-103

14 Guyatt GH, Oxman AD, Kunz R, *et al.* GRADE guidelines: 2. Framing the question and deciding on important outcomes. *J Clin Epidemiol*. 2011;64:395–400. doi: 10.1016/J.JCLINEPI.2010.09.012

15 Kirkham JJ, Davis K, Altman DG, *et al.* Core Outcome Set-STAndards for Development: The COS-STAD recommendations. *PLOS Med*. 2017;14:e1002447. doi: 10.1371/journal.pmed.1002447

16 Gargon E, Gorst SL, Harman NL, *et al.* Choosing important health outcomes for

comparative effectiveness research: 4th annual update to a systematic review of core outcome sets for research. *PLoS One*. 2018;13:e0209869. doi: 10.1371/JOURNAL.PONE.0209869

17 Gargon E, Gorst SL, Matvienko-Sikar K, *et al.* Choosing important health outcomes for comparative effectiveness research: 6th annual update to a systematic review of core outcome sets for research. *PLoS One*. 2021;16:e0244878. doi: 10.1371/JOURNAL.PONE.0244878

18 Gorst SL, Gargon E, Clarke M, *et al.* Choosing Important Health Outcomes for Comparative Effectiveness Research: An Updated Review and Identification of Gaps. *PLoS One*. 2016;11:e0168403. doi: 10.1371/JOURNAL.PONE.0168403

VERSION 2 - REVIEW

**Reviewer**	**1**
**Name**	**Bui, Michael**
**Affiliation**	**University of Twente, Health Technology and Services Research**
**Date**	**04-Aug-2026**
**COI**	

I appreciate the opportunity to review this manuscript again. In particular, I would like to thank the authors for addressing each of my concerns so extensively and thoughtfully. The unclarities I raised in the introduction have been alleviated, and the reporting of the methodology has improved substantially. Specifically, the authors:

1. clarified how their contributions may aid HTA of shared decision-making interventions

2. elaborated on their literature search and subsequent data extraction procedures for initial COS conceptualisation

3. improved the transparency of their sample size justification for qualitative interviews

4. clarified the selection criteria used for purposive sampling of experts

5. addressed issues of ceiling effects for defining consensus thresholds

6. justified their choice to modify their Delphi study design by relying on a nominal group technique rather than survey in the final round

The authors have been highly responsive to reviewer feedback and I do not foresee any other concerns.
